# Risk prediction models for post-stroke cognitive impairment: a systematic review and meta-analysis

**DOI:** 10.3389/fneur.2026.1798991

**Published:** 2026-08-27

**Authors:** Changsen Li, Xiangui Lv, Xiaohui Wang, Xiaomei Xu, Daiqiang Liu, Xinwei Chen, Dongjiao Dou, Liuna Lin, Lin Chen, Chao Huang, Lvlin Chen

**Affiliations:** 1 Department of Intensive Care Medicine, Affiliated Hospital of Chengdu University, Chengdu, Sichuan, China; 2 Department of Nursing, Affiliated Hospital of Chengdu University, Chengdu, Sichuan, China; 3 Chengdu University, Chengdu, Sichuan, China

**Keywords:** cognitive impairment, meta-analysis, post-stroke, risk prediction models, stroke-diagnosis

## Abstract

**Background:**

The number of risk prediction models for post-stroke cognitive impairment has been increasing, but the prediction performance and clinical applicability of existing models still require further verification.

**Objective:**

To systematically evaluate the published risk prediction models for cognitive impairment after stroke in patients with acute stroke.

**Methods:**

The literatures on the risk prediction model of cognitive impairment after stroke in PubMed, EMBASE, web of science and Cochrane library databases on October 1, 2025 were retrieved. Extract data from the selected studies, including the collected data including author, publication year, country, time of inclusion, whether it is a multicenter study, research type, stroke type, diagnostic criteria for cognitive impairment, number of post-stroke cognitive impairment cases, model development method, validation method, missing data processing, model presentation, model prediction performance, model calibration, and predictive factors finally included in the model. The Prediction Model Risk of Bias Assessment Tool checklist was used to assess the risk of bias and applicability.

**Results:**

A total of 4,715 studies were retrieved and 29 studies were included after the selection process. These studies were published from 2016 to 2023. In 29 studies, the area under the curve of development set models ranged from 0.69 to 0.97. Using random-effects meta-analysis, the descriptive summary AUC across 12 development set models was 0.85 (95% CI: 0.80–0.90), with substantial heterogeneity (I^2^ = 93.6%). For the 7 validation models, the descriptive summary AUC was 0.82 (95% CI: 0.76–0.87, I^2^ = 71.2%). Given the considerable clinical and methodological heterogeneity across studies, these pooled estimates should be interpreted as a descriptive summary of the reported AUC distribution rather than as a precise estimate of a single underlying predictive performance.

**Conclusion:**

Although the descriptive summary AUC values (0.85 for development, 0.82 for validation) suggest apparently acceptable discriminative ability, all 29 included prediction model studies were judged to be at high overall risk of bias according to the PROBAST tool (particularly in the analysis domain). This likely leads to overestimation of reported model performance. Therefore, the models should be considered as preliminary tools that require further rigorous external validation before clinical application. Future research should develop a risk prediction model with larger samples and multi centers, and carry out internal and external validation.

**Systematic review registration:**

https://www.crd.york.ac.uk/prospero/search, identifier: CRD42024502063.

## Introduction

Stroke is a serious disease, the third leading cause of death and long-term disability in the world, and has caused a huge burden worldwide (1, 2). Poststroke cognitive impairment (PSCI) is a clinical syndrome characterized by cognitive impairment that occurs after stroke (3). About 44% of individuals have cognitive impairment within 6 months after stroke (4). The incidence of PSCI in the first year after stroke was as high as 60% (5). PSCI not only seriously harms the physical and mental health of patients, affects the quality of life, but also increases the economic burden of patients’ families. Early after stroke, cognitive impairment is reversible. Early identification and intervention of PSCI can delay the progress of cognitive impairment and even reverse the cognitive results (6). Early identification of high-risk patients with cognitive impairment and cognitive intervention for high-risk patients are of great significance to improve the clinical outcomes of patients.

The risk prediction model of PSCI includes combining multiple predictors to estimate the probability or risk of post-stroke cognitive impairment (7). In recent years, some scholars have constructed relevant prediction models, but the prediction performance requires further validation prediction model Therefore, this study explored and systematically evaluated the relevant literature of PSCI risk prediction model, in order to provide the basis for the application and optimization of PSCI risk prediction model.

## Methods

### Inclusion and exclusion criteria

*Inclusion criteria*

Patients were aged ≥18 years; The research content is the risk prediction model of post-stroke cognitive impairment and describes the process of model establishment, validation and evaluation; Research types include cohort study, case–control study and cross-sectional study.

*Exclusion criteria*

It is only a study of risk factors, and there is no process or method to build the model; Research on the accuracy of single factor prediction model; Guidelines, expert opinions, reviews and animal studies; The original text cannot be obtained or the data is incomplete.

### Literature research strategy

PubMed, EMBASE, Web of Science, and the Cochrane Library were systematically searched for studies on predictive models for post-stroke cognitive impairment, covering the period from database inception to October 1, 2025. A combination of controlled vocabulary (MeSH) and free-text keywords was used. The search terms included “stroke,” “brain infarct,” “cerebral hemorrhage,” “cognitive impairment,” “cognitive dysfunctions,” “prediction model,” “prediction tool,” and “diagnostic model.” The full search strategy is detailed in Supplementary material 1.

### Literature screening and data extraction

Two researchers (XL and XC) independently conducted literature screening and data extraction, and then cross checked the results. For the controversial literature, the third senior researcher (CH) was invited to make a judgment. All extracted data items were collected according to the Checklist for Critical Appraisal and Data Extraction for Systematic Reviews of Prediction Modelling Studies (CHARMS) (8). The data collected include the author, year of publication, country, whether it is a multicenter study, type of study, type of stroke, diagnostic criteria for PSCI, number of PSCI cases, model development method, validation method, missing data handling, model presentation, model prediction model performance, model calibration, and predictive factors finally included in the model.

### Risk of bias assessment

The risk of bias assessment was carried out independently by two researchers (XL and XC), and any discrepancies were resolved by a third researcher (CH). The Prediction model Risk Of Bias ASsessment Tool (PROBAST) was used to evaluate the risk of bias (7, 8). PROBAT is a checklist designed for systematic review of diagnostic or prognostic prediction models. Risk of bias assessment includes participants, predictors, outcomes, and analysis.

### Data synthesis and statistical analysis

For meta-analysis of AUC values, we required that studies report AUC with either standard error (SE) or 95% confidence interval (CI). If only a point estimate was provided without SE/CI, the study was excluded from quantitative synthesis but remained in the narrative review. Among the 29 included studies, 17 development models and 22 validation models were excluded from the meta-analysis due to incomplete reporting of SE/CI. We did not contact the original study authors to request missing variance estimates, as the primary aim of this review was to synthesize available published data and to highlight reporting deficiencies in the current literature. Stata 14.0 software was used for meta-analysis of the predictive AUC value of predictive factors in the model. The DerSimonian-Laird estimator was used for random-effects meta-analysis. Firstly, Q-test was used tothe 12 modeling models included determine whether there was heterogeneity between studies. If *p* > 0.1 or I^2^ < 50%, it was considered that there was good homogeneity between studies, and a fixed effects model was used for analysis; If *p* < 0.1 or I^2^ > 50%, heterogeneity between studies is considered and a random effects model is used for analysis.

## Results

### Study selection

**Figure 1:**
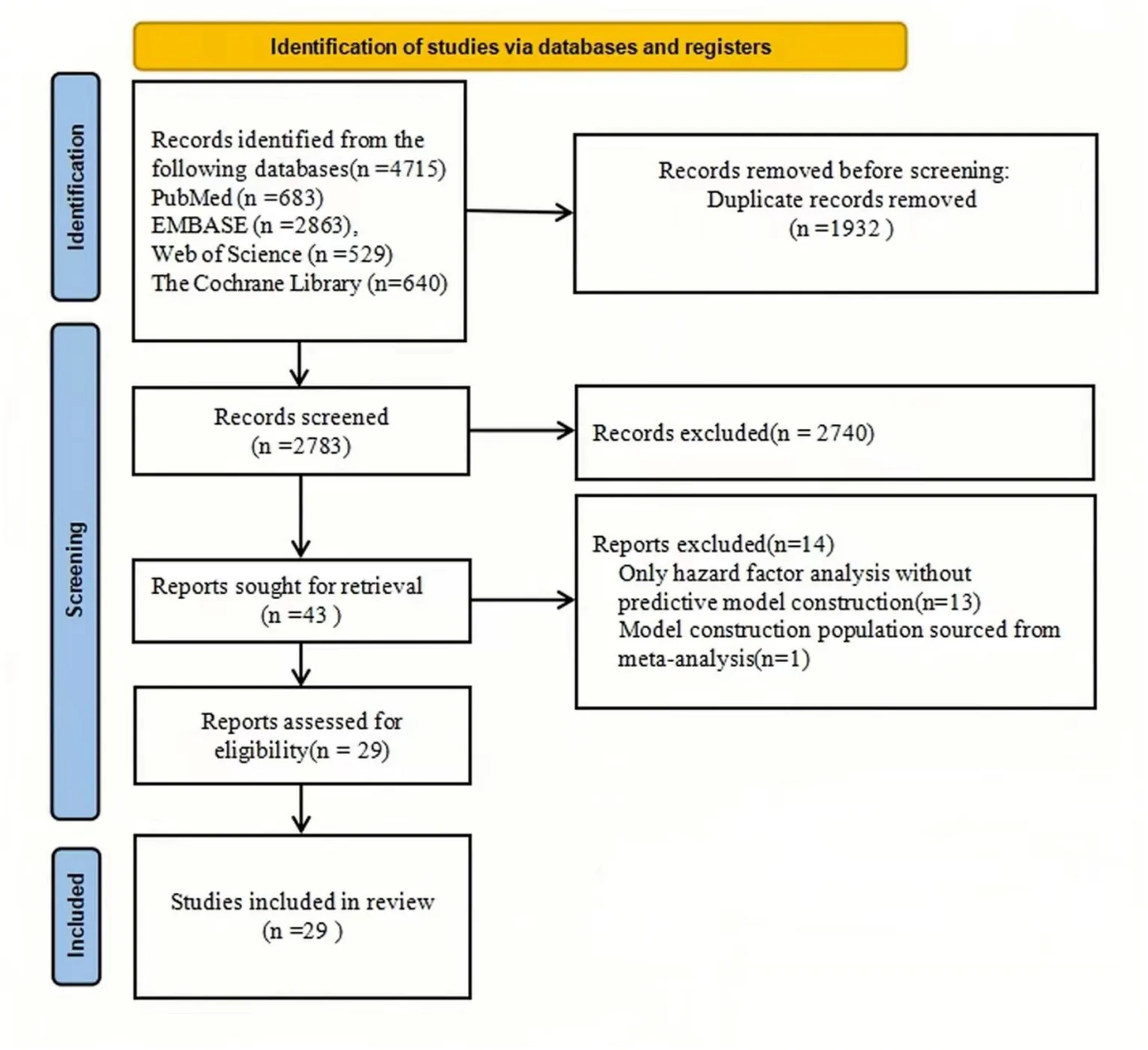
Flowchart of study selection.

A total of 4,715 relevant literatures (PubMed = 683, EMBASE = 2,863, web of science = 529, Cochrane Library = 640) were obtained in the preliminary examination. After screening, 29 literatures were finally included. The process and results of literature screening were shown in Figure 1.

### Basic characteristics of literature

This systematic review identified 29 PSCI prediction models, with most studies originating from Asia and using logistic regression as the primary modeling method. Among the included studies, there were 13 multicenter studies, 8 were retrospective cohort studies, 15 were prospective cohort studies, 5 were retrospective case–control studies, and 1 was a cross-sectional study (see Table 1).

**Table 1:** Characteristic of the included studies.

First author	Year	Country	Multicenter	Study design	Follow-up duration	Stroke type	Diagnostic criteria for cognitive impairment
Aamodt et al. (11)	2021	Norway	Yes	Prospective cohort study	3 m	Stroke	DSM−5
Betrouni et al. (28)	2022	Germany	Yes	Prospective cohort study	6,12 m	Stroke	MMSE score<27 or MoCA score<25
Chander et al. (29)	2017	Singapore	Yes	Retrospective cohort study	3–6 m	IS	IQCODE≥3.6
Ding et al. (30)	2019	China	No	Prospective cohort study	6-12 m	AIS	MoCA, MMSE, DSM-5
Dong et al. (31)	2021	China	Yes	Retrospective case–control study	6 m	AIS	MoCA < 22
Gong et al. (32)	2021	China	No	Prospective cohort study	6 m, 12 m	IS	MoCA < 22
Gu et al. (33)	2022	China	No	Prospective cohort study	6 m	Intracerebral hemorrhage	MoCA<26
Hbid et al. (12)	2021	Britain	Yes	Retrospective cohort study	3 m, 12 m, 5 y	First occurrence of cerebral stroke	MMSE <24 or AMT < 8
Wencan Ji (13)	2023	China	No	Prospective cohort study	3 m,6 m	AIS	MoCA<23
Kandiah et al. (7)	2016	Singapore	No	Retrospective cohort study	3 m,6 m	Mild Stroke	MMSE≤25 or MoCA ≤22
Lee et al. (14)	2023	South Korea	No	Retrospective cohort study	3 m-6 m	AIS	MMSE<24
Lee et al. (15)	2023	South Korea	No	Retrospective case–control study	3 m	AIS	MoCA, MMSE
Lee et al. (34)	2022	South Korea	No	Prospective cohort study	3 m,6 m	Acute cerebral infarction or transient ischemic attack	MMSE, CDR
Ling et al. (16)	2020	China	No	Prospective cohort study	3 m	IS	junior school education level or above,: MoCA<26; primary school education: MoCA < 21; illiterate patients: MoCA < 15;
Lopes et al. (17)	2021	France	Yes	Retrospective cohort study	6 m,36 m	IS	IQCODE 49 ± 2
Ma et al. (9)	2022	China	No	Retrospective cohort study	-	Type 2 diabetes patients with AIS	mild cognitive impairment: MoCA<26 severe cognitive impairment: MoCA<20
Munthe-Kaas et al. (35)	2022	Norway	Yes	Prospective cohort study	3 m	Cerebral stroke	Premorbid cognitive status based on GDS
Lim et al. (18)	2017	South Korea	Yes	Prospective cohort study	3 m	IS	AHA/ASA Scientific Statement vascular dementia criteria, which requires deficits in at least two cognitive domains
Röhrig et al. (19)	2022	Germany	Yes	Prospective cohort study	3 m	Acute right hemispheric stroke	bells cancelation test the Center of Cancelation
Shang et al. (36)	2022	China	No	Prospective cohort study	3 m,6 m, 12 m	Mild AIS	MOCA < 22
Tao et al. (10)	2022	China	Yes	prospective cross-sectional study	—	Cerebrovascular disease;	MMSE
Vlachos et al. (37)	2023	Norway	Yes	Retrospective cohort study	12 m	Mild acute stroke	The Barthel ADL index and mRS
Weaver et al. (20)	2023	Netherlands	Yes	Retrospective cohort study	15 m	Cerebral stroke	MoCA
Ye et al. (25)	2022	China	No	Retrospective case–control study	3 m	Lacunar infarction	MMSE <24
Yuan et al. (21)	2021	China	No	Retrospective case–control study	0-12 m	Stroke	MMSE <24, MOCA≤26
Zha et al. (38)	2021	China	No	Retrospective case–control study	3 m	Stroke	MMSE
Zhu et al. (22)	2020	China	No	Prospective cohort study	3 m,6 m	IS	MMSE, MoCA
Zhu et al. (39)	2022	China	Yes	Prospective cohort study	3 m	AIS	MMSE <25
Zhu et al. (27)	2019	China	Yes	Prospective cohort study	3 m	IS	MMSE<27, MoCA<25

AIS, acute ischemic stroke; IS, ischemic stroke; “—,” not reported; DSM−5, Diagnostic and Statistical Manual of Mental Disorders; MMSE, mini-mental state examination; MoCA, Montreal cognitive assessment; AMT, Abbreviated Mental Test; mRS, modified Rankin Scale; CDR, clinical dementia rating; GDS, global deterioration scale; IQCODE, informant questionnaire on cognitive decline in the elderly; AHA, American Heart Association; ASA, American Stroke Association; ADL, activities of daily living.

### Risk of bias assessment

**Figure 2:**
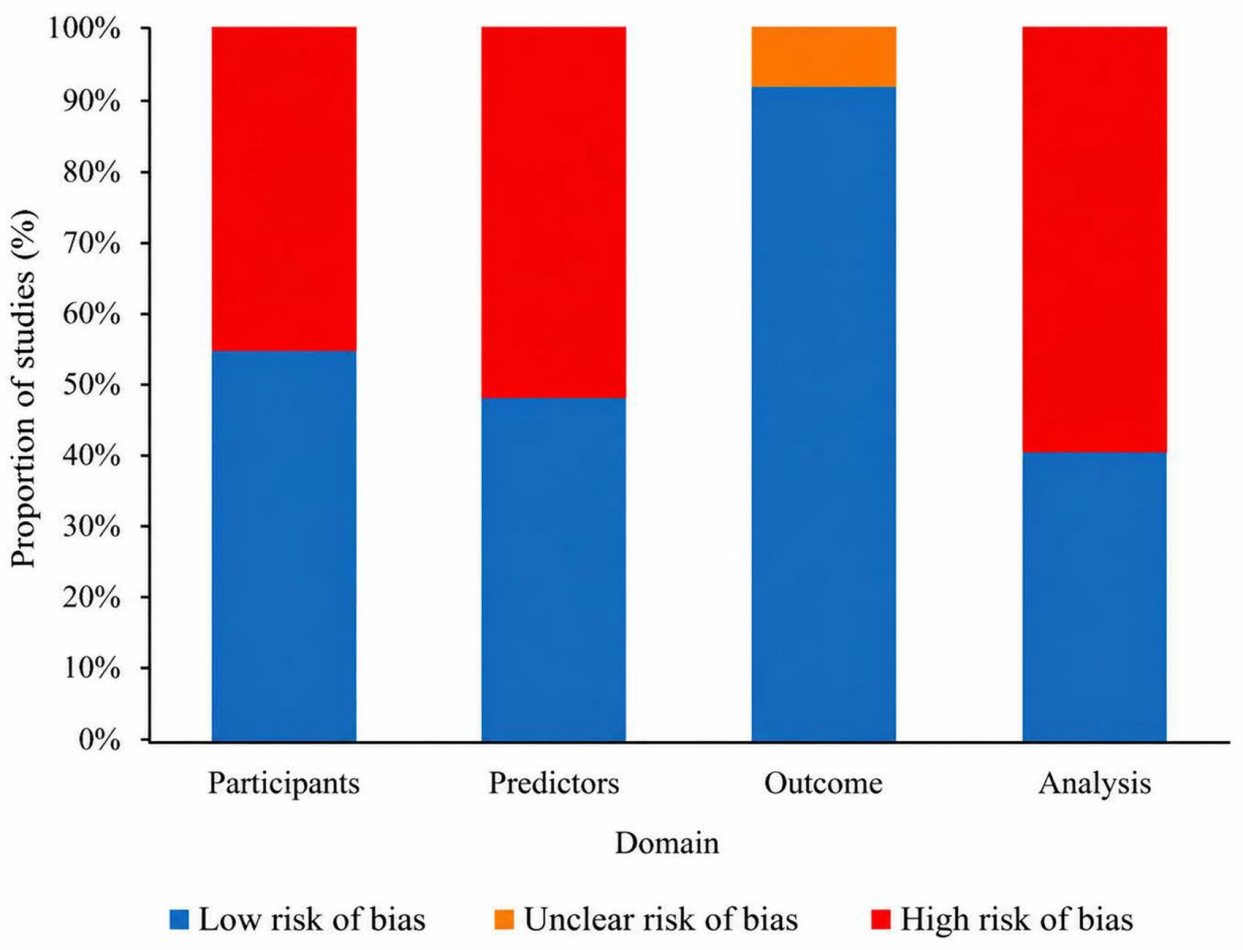
Risk of bias assessment.

In the assessment of Participants, 16 prospective studies were rated as low risk, while 13 retrospective studies were rated as high risk. The primary reason for the high-risk rating in retrospective studies was that key predictors related to cognitive impairment might not have been routinely collected in medical records, or were measured in a non-standardized manner across different sites, introducing potential selection and information bias. In the assessment predictors, 14 studies were rated as low risk and 15 as high risk. The main reason is that the data of these 15 studies were from multiple centers, and there may be differences in their data acquisition and evaluation, resulting in bias. In assessment of outcome, 2 studies (9, 10)were considered unclear risk, mainly because the time of PSCI assessment was not reported.17 studies were considered to be at high risk of bias in the assessment of analysis. The main reason was that univariate analysis was used to screen predictors, and Brier score, calibration curve and Hosmer-Lemeshow were not evaluated. Only 2 studies described methods for handling missing data (11, 12), while the remaining studies did not indicate whether appropriate statistical methods were chosen for handling missing data. Missing data may lead to bias in the association between influencing factors and results. Only 12 models did not use univariate analysis to screen predictive factors (10–21). Screening predictive factors based on the results of single factor analysis is a common practice for most predictive model development, but it can lead to the loss of effective predictive factors due to issues such as multicollinearity between independent variables, which is also one of the important reasons for high model bias. The risk of bias assessment of the included studies is shown below in Figure 2.

### Information of the included prediction models

Among the included studies, 21 studies used logistic regression analysis to develop prediction models. Machine learning methods were also used, such as Support Vector Machine (SVM), Random Forest (RF), Gaussian Naive Bayes (GNB), Extreme Gradient Boost (XGBoost), K Nearest Neighbor (KNN), Adaptive Boosting (AdBoost), Multilayer Perceptron(MLP), Light Gradient Boosting Machine (LightGBM), and Neural Network(ANN), Classification And Regression Tree(CART). Fourteen studies were validated internally and eight studies were validated externally. The most commonly used predictors in all models were age, years of education and diabetes, which appeared in 17, 11 and 6 models, respectively. Other commonly used predictors included the National Institutes of Health Stroke Scale (NIHSS) admission score and gender, which were used in 5 and 4 models, respectively. Table 2 presents the included model information.

**Table 2:** Overview of the information of the included prediction models.

First author	Development model PSCI cases	Validation method	Model development method	Missing data handling	Model presentation	Model performance	Calibration method	Final predictors
Aamodt et al. (11)	62/227 (27%)	internal validation	Classifier model	Complete case analysis	Script	A:0.88	—	Stroke lesion volume WMH volume Left occipital and temporal cortical thickness Right cingulate cortical thickness NIHSS Antiplatelet medication intake Education
Betrouni et al. (28)	77/327 (24%)	internal validation	random forest	—	Script	A: 0.90 ± 0.03 B: 0.77.	—	Structural MR images (T1-w)
Chander et al. (29)	78/209(37%)	internal validation,external validation	logistic regression	—	The risk scoring formula obtained through the regression coefficients of each factor	A:0.82 (0.76–0.88) B: internal validation 0.78 (0.71–0.85); external validation 0.75 (0.71–0.79)	Brier score	Chronic lacunes Hyperintensities in white matter regions Age Non - lacunar cortical infarct Global cortical atrophy Education
Ding et al. (30)	77/145 (53%)	-	Logistic regression	—	The risk scoring formula obtained through the regression coefficients of each factor	A:0.88 (0.83–0.94)	—	Age Periventricular hyperintensity grading Diabetes mellitus The number of acute nonlacunar infarcts
Dong et al. (31)	131/383 (34%)	internal validation,external validation	logistic regression	—	—	A:0.93 B: 0.76 (0.66–0.85).	Hosmer-Lemeshow	Diabetes NIHSS Education Age MoCA LDL-C level
Gong et al. (32)	122/228 (54%)	internal validation,external validation	Logistic regression	—	Nomogram model	A:0.83 B:0.81	Hosmer-Lemeshow, calibration curve	Age Female Fazekas score Education Number of intracranial atherosclerotic stenosis (ICAS) HbA1c Cortical infarction.
Gu et al. (33)	69/123 (56%)	external validation	Logistic regression	—	Nomogram model	A:0.94(0.89–0.99) B:0.91	Calibration curve	Age Education Hematoma volume Dominant-hemisphere hemorrhage Hemoglobin mean corpuscular volume RBC distribution width
Hbid et al. (12)	1000/2468 (41%)	internal validation	Logistic regression	Calculate missing values multiple times to reduce bias	-	A: 3 m:0.88 (0.85–0.90) 1y:0.90 (0.86–0.92) 5y:0.87 (0.85–0.91) B:0.73	Calibration curve, Brier score	Age Sex Ethnic group Cognition score at the onset of stroke Barthel-index score at baseline Glasgow coma scale Stroke subtype (LACI, PACI, POCI, TACI) Diabetes Left hemisphere stroke Dysphasia
Wencan Ji (13)	102/331 (31%)	external validation	GNB, XGBoost, Logistic. Random Forest, SVM, KNN, AdBoost, MLP, LightGBM	—	Script	A:0.94 (0.91–0.97) B:0.92 (0.85–0.99)	calibration curve, Brier score	Age Education NIHSS Cerebral white matter degeneration Homocysteine C-reactive protein
Kandiah et al. (7)	78/209 (37)	internal validation,external validation	logistic regression	—	The risk scoring formula obtained through the regression coefficients of each factor	A:0.83 (0.77–0.88) B:0.78 (0.70–0.85)	calibration curve	Age Education Acute cortical infarcts White matter hyperintensity Chronic lacunes Global cortical atrophy Intracranial vascular stenosis
Lee et al. (14)	290/951 (30%)	—	logistic regression, SVM, XGBoost, and ANN	—	Script	A: XGBoost: 0.79 (0.68–0.89) ANN: 0.74 (0.62–0.84) SVM:0.72 (0.59–0.83) LR:0.71 (0.59–0.83) -	—	Age Stroke severity Stroke volume Mesial temporal lobe atrophy Fasting blood glucose Cortical lesions.
Lee et al. (15)	NA/87	internal validation	Ridge, Lasso, ElasticNet, SVM, and AdaBoost regression	—	-	The predictive powers (R^2^) were 0.76 and 0.65 for the left and right stroke groups, respectively.	—	MoCA EEG-based network properties(higher global efficiency, clustering coefficient, and lower characteristic path length)
Lee et al. (34)	19/110 (17%)	internal validation	Logistic regression	—	Script	A:0.94 (0.89–0.98) B:0.75 (0.64–0.85)	—	Age Positron emission tomography (PET) image Neutrophil–lymphocyte ratio Platelet-lymphocyte ratio Body mass index
Ling et al. (16)	53/93 (57%)	—	Multivariate logistic regression	—	The risk scoring formula obtained through the regression coefficients of each factor	A:0.86(0.77–0.94)	—	NIHSS Brain atrophy Stroke recurrence Leukoaraiosis Homocysteine Abundance of Enterobacteriaceae
Lopes et al. (17)	25/72 (35)	internal validation,external validation	Ridge regression	—	Script	Predict memory, attention, visuospatial functions, and language functions 36 months poststroke (r2: 0.67, 0.73, 0.55). -	—	Functional networks measured using MRI
Ma et al. (9)	94/161 (58%)	—	Logistic regression	—	The risk scoring formula obtained through the regression coefficients of each factor	A:0.97	—	Sex Age Education Recurrent cerebral infarction Course of diabetes and serum albumin
Munthe-Kaas et al. (35)	91/598 (15%)	—	Logistic regression	—	-	A:0.76	—	Frailty MRS
Lim et al. (18)	50/308 (16%)	—	Logistic regression	—	The risk scoring formula obtained through the regression coefficients of each factor	A:0.74	—	Age Women More severe stroke Had history of premorbid cognitive decline
Röhrig et al. (19)	27/103 (26%)	—	SVM	—	Script	—	—	White matter hyperintensities
Shang et al. (36)	253/454 (56%)	—	Logistic regression	—	The risk scoring formula obtained through the regression coefficients of each factor	A:0.72	—	Age History of stroke DMTS of the infarct Anterior circulation cerebral infarction NLR
Tao et al. (10)	204/302 (67%)		Ordinal logistic regression.			A: mVCI: 0.69 sVCI:0.70	—	Erythrocytopenia
Vlachos et al. (37)	21/117	—	Logistic regression		The risk scoring formula obtained through the regression coefficients of each factor	—	—	Age Not working prior to stroke CT or MRI finding of multiple stroke lesions
Weaver et al. (20)	1286/2950	Internal validation	Logistic regression		The risk scoring formula obtained through the regression coefficients of each factor	A:0.88	calibration plot	Infarcts in the left frontotemporal lobes Left thalamus Right parietal lobe
Ye et al. (25)	38/219	Internal validation	Logistic regression	—	Nomogram model	A:0.86 (0.79–0.93) B:0.81 (0.69–0.94)	Hosmer-Lemeshow H test	Age Diabetes White blood count Cystatin C Serum amyloid A
Yuan et al. (21)	118/376	Internal validation,external validation	LASSO regression	—	Nomogram model	B: internal validation: 0.89 (0.82–0.91) external validation: 0.85 (0.83–0.91)	Calibration plot	Education Diabetes Left frontal NAA/Cr Left thalamus NAA/Cr Left hippocampus NAA/Cr
Zha et al. (38)	87/367	Internal validation	Logistic regression	—	Nomogram model	A:0.81 B:0.82	Calibration plot	Sex Age NIHSS NLR
Zhu et al. (22)	66/104	—	Logistic regression, CART	—	A preliminary model is constructed by using the risk scoring formula derived from the regression coefficient of each factor, and then the relative importance is ranked by CART method and a prediction model is validation d	A:0.82	—	Education BMI MoCA MMSE
Zhu et al. (39)	228/599	—	Logistic regression	—	The risk scoring formula obtained through the regression coefficients of each factor	A:0.69 (0.65–0.73)	Hosmer-Lemeshow	Age Sex Education BMI Current smoking Alcohol drinking Time from onset to randomization, Diastolic blood pressure, NISS Ischemic stroke subtype Randomized treatment Medical history (hypertension, diabetes, hyperlipidemia, coronary heart disease and history of stroke) Use of antihypertensive Medications hsCRP and homocysteine sTREM2.
Zhu et al. (27)	298/638	—	Logistic regression	—	The risk scoring formula obtained through the regression coefficients of each factor	A: 0.73 (0.69–0.77)	—	Rheumatoid factor Matrix metalloproteinase-9 Total homocysteine

A, development set; B, validation cohort; ICAS, intracranial atherosclerotic stenosis; MRS, Modifed Rankin Scale; NISS, National Institutes of Health Stroke Scale; MMSE, Mini mental state examination scale; MoCA, Montreal Cognitive Assessment; hsCRP, High sensitivity C-reactive protein; BMI, Body mass index; NLR, Neutrophil-lymphocyte ratio; DMTS, diameter of maximum transverse section.

### Model performance and results

**Figure 3:**
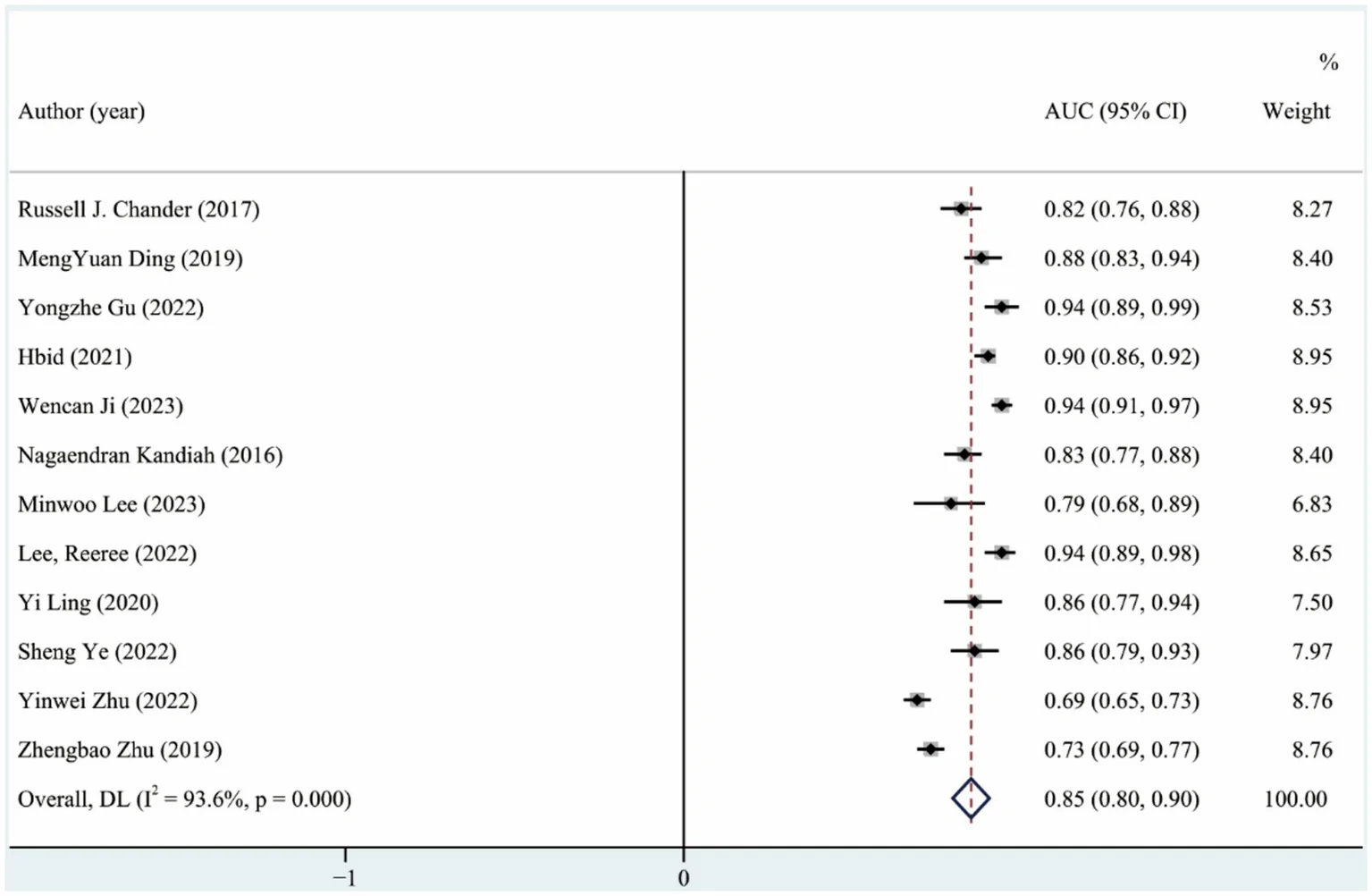
Forest plot of the random effects meta-analysis of pooled AUC estimates for 12 development set models.

**Figure 4:**
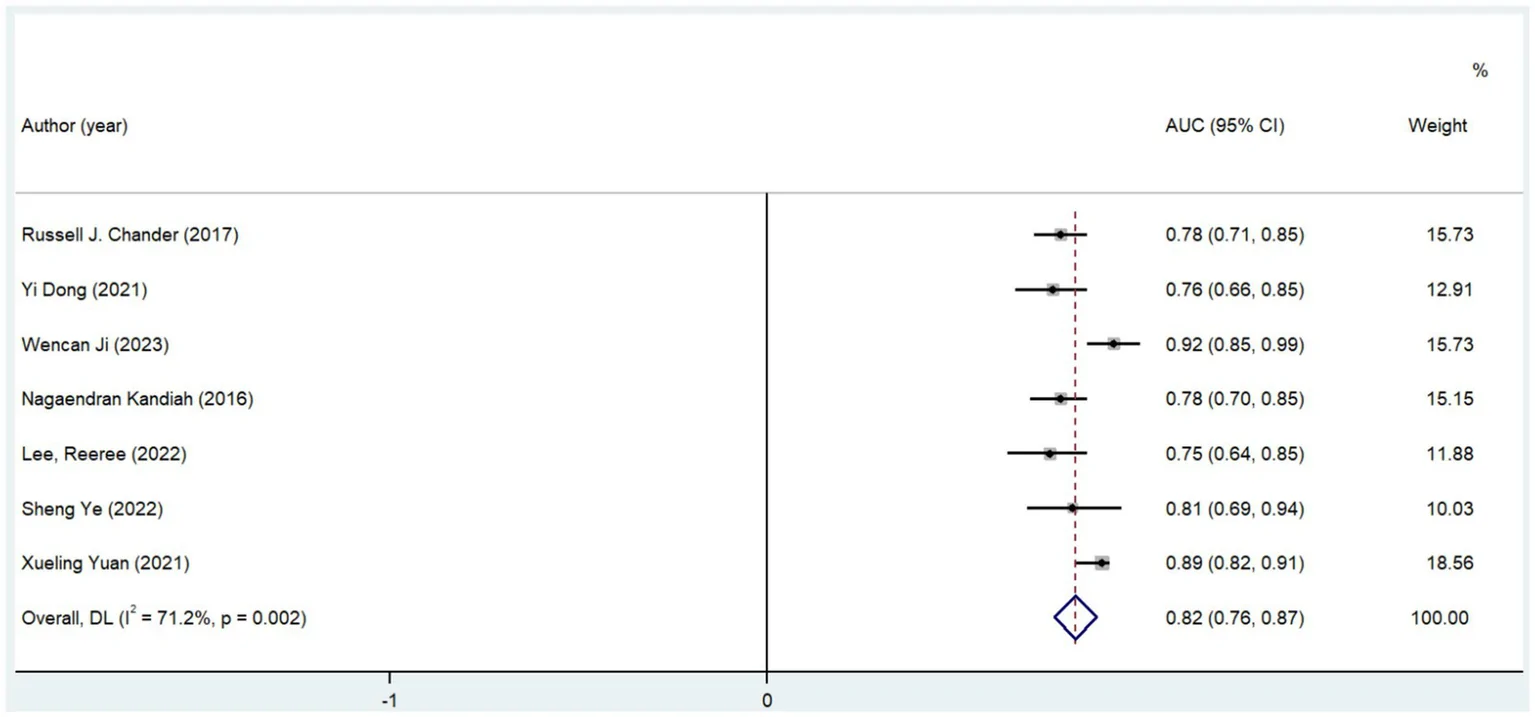
Forest plot of the random effects meta-analysis of pooled AUC estimates for 7 validation models.

The AUC value of the development set for the predictive model is reported to be between 0.69 to 0.97. The AUC value of the report prediction model validation set is between 0.73 to 0.92. Of the 29 included studies, only 12 development set models and 7 validation models reported AUC with SE or 95% CI, thereby meeting the criteria for inclusion in quantitative synthesis. The remaining studies were excluded from the meta-analysis due to incomplete reporting of variance estimates. Among them, studies by Ji et al. (13) and Lee et al. (14) involved multiple model development methods based on the same cohort; therefore, only the model with the optimal performance from each study was included in the meta-analysis. In the model development set, the I^2^ value was 93.6% (*p* < 0.001), indicating that there was a high degree of heterogeneity among the studies. The AUC of the combined modeling model was calculated by using the random effect model, and the result value was 0.85 (95% CI: 0.80–0.90) (Figure 3). In the model validation set, the I^2^ value was 71.2% (*p* = 0.002), indicating that there was a high degree of heterogeneity among the studies. The AUC of the combined validation model was calculated using the random effect model, and the result value was 0.82 (95% CI: 0.76–0.87) (Figure 4). Due to the limited number of studies reporting complete data for subgroup analyses, we did not perform meta-regression or formal subgroup analyses. This is a limitation of the current review.

### Sensitivity analysis

Leave-one-out sensitivity analyses were performed to assess the robustness of the meta-analysis results. For the 12 development set models (Supplementary material 3), the pooled AUC estimates after sequentially omitting each study ranged from 0.83 to 0.90, all encompassing the original estimate of 0.85. For the 7 validation set models (Supplementary material 3), the estimates ranged from 0.76 to 0.87, all consistent with the original estimate of 0.82. No single study disproportionately influenced the summary estimates, supporting the robustness of the descriptive findings despite substantial heterogeneity.

## Discussion

Stroke is still one of the critical cardiovascular diseases in the world. Once PSCI occurs, it is difficult to reverse, and the diagnosis of PSCI needs 3–6 months after stroke. Therefore, early identification and prevention are essential. PSCI risk prediction models have the potential to support early detection and prevention. However, due to the high risk of bias identified in this review, their current predictive performance is likely overestimated, and their readiness for clinical implementation remains limited. In recent years, the research on the development of its prognosis model has gradually increased, but the quality of the research needs to be evaluated.

Twenty two models were analyzed by logistic regression. Ji et al. (13) compared eight machine learning algorithms (GNB, XGBoost, LR, RF, SVM, KNN, AdBoost, MLP, LightGBM) through comprehensive analysis, Consider the GNB model as the optimal model. Lee et al. (14) used four machine learning algorithms (LR, SVM, XGBoost, and ANN) for comparison, with XGBoost having the highest average AUC value, followed by SVM, ANN, and logistic regression analysis. Lee et al. (15) compared five machine learning algorithms (Ridge, Lasso, ElasticNet, SVM, and AdaBoost regression.), among which Ridge had the strongest predictive ability, while the left and right stroke groups had predictive abilities (R^2^) of 0.76 and 0.65. Zhu et al. (22) applied two modeling methods, logistic regression and CART, and the results showed that both methods performed well. The CART model led to some improvements in the predicted values and provided a ranking for risk factors related to PSCI. Some included studies reported improved discrimination using machine learning approaches compared with traditional logistic regression within the same derivation cohorts. However, evidence across clinical prediction research suggests that the relative performance of machine learning versus regression-based methods is highly context-dependent, influenced by factors such as sample size, number of predictors, data quality, and the extent of internal and external validation (23). These findings should be interpreted cautiously, as superior performance in a single derivation cohort does not guarantee generalizability.

The performance of clinical prediction models mainly includes discrimination and calibration. Most studies use AUC to evaluate the discriminative power of models. Among the 29 studies included, most prediction models reported an AUC > 0.7, suggesting apparently good discriminative ability. However, given that all included studies were judged to be at high risk of bias, this apparent performance is likely overestimated. AUC should be interpreted in conjunction with calibration measures and the results of external validation. In terms of calibration, 5 studies used calibration curves, 4 studies used Hosmer-Lemeshow tests, and 3 studies used calibration plots to evaluate model calibration. Some studies suggest that the *p*-value obtained from the Hosmer-Lemeshow test cannot be used to measure calibration accuracy. It is recommended to use Brier score, as the lower the Brier score, the better the calibration accuracy (24). The exclusion of more than half of the studies from meta-analysis due to incomplete reporting may introduce selection bias. Studies with more complete reporting might differ systematically from those excluded, potentially leading to overestimation of the pooled AUC. AUC alone is insufficient to judge a model’s clinical usefulness. Calibration – the agreement between predicted and observed risks – was poorly reported; over half of the studies did not report any calibration measure. Moreover, no study performed decision curve analysis to assess net benefit. Future prediction model studies should adhere to the TRIPOD statement, reporting both discrimination and calibration, and ideally include decision curve analysis.

The PROBAST assessment revealed that all 29 studies had a high overall risk of bias, especially in the analysis domain. Common methodological flaws included: (1) predictor selection based on univariate analysis, which may omit important variables; (2) lack of appropriate handling of missing data (only 2 studies reported methods); (3) insufficient or absent calibration assessment (e.g., Brier score, calibration plots). These issues are known to inflate apparent model performance due to overfitting and optimism. Consequently, the reported AUC values likely overestimate the true discriminative ability when applied to new populations. Readers should interpret the model performance with caution, and clinical uptake should not be based solely on these results.

According to the PROBAST checklist, all 29 included prediction model studies were judged to have a high overall risk of bias, which limits the practical utility of these predictive models. The heterogeneity between different models is relatively high, which may be related to the differences in population, predictive factors, and research methods among different models. The research issues mainly focus on methodology and statistical analysis, with 15 studies from a single center and 14 studies choosing to use retrospective data.

The most frequently included predictors—age, years of education, and diabetes—have well-established pathophysiological links to PSCI. Age is associated with reduced brain reserve and increased vulnerability to vascular and neurodegenerative changes following stroke. Lower education may reflect lower cognitive reserve, which reduces the ability to compensate for stroke-induced brain damage. Diabetes contributes to PSCI through multiple mechanisms, including chronic hyperglycemia, microvascular damage, and enhanced neuroinflammation. In recent years, biomarkers have become a research hotspot for predicting PSCI, with important predictive factors for the risk of PSCI being gut microbiota (16), serum amyloid A (SAA) (25), blood uric acid levels (26), rheumatoid factors, matrix metalloproteinase-9, and total homocysteine (27). However, a single biomarker may not be sufficient, and it is necessary to combine multiple biomarkers for diagnosis to improve the accuracy of diagnosing PSCI, thereby facilitating early targeted treatment of PSCI.

### Limitations

This review has several potential limitations. First of all, the overrepresentation of studies from China and other Asian countries raises concerns about external validity to Western populations. Most included studies did not report stroke subtype according to the TOAST classification, and hemorrhagic stroke was often reported without specifying the type. This limits our ability to examine whether prediction model performance differs across stroke etiologies. Moreover, healthcare systems and post-stroke management protocols vary significantly across regions, which could affect the incidence and detection of PSCI. Therefore, existing models should be externally validated in diverse ethnic and geographical cohorts before generalizing their use. Future research should also consider developing or recalibrating models for specific populations.

## Conclusion

In summary, this systematic review found that while existing PSCI prediction models show apparently good discriminative ability (pooled AUC 0.85 and 0.82 for development and validation sets, respectively), all included studies had a high risk of bias. Therefore, the reported predictive performance is likely overestimated, and current models are not yet ready for routine clinical use. Future research should adhere to rigorous methodological standards, including large multi-center prospective cohorts, appropriate missing data handling, and both internal and external validation with careful assessment of calibration and clinical utility.

## Data Availability

The original contributions presented in the study are included in the article/Supplementary material, further inquiries can be directed to the corresponding authors.
