# Supplementary material for: Risk prediction models for post-stroke cognitive impairment: a systematic review and meta-analysis
Source: Front Neurol. 2026 Aug 27;17:1798991. doi: 10.3389/fneur.2026.1798991 (PMC13558108; doi:10.3389/fneur.2026.1798991)
Supplement: Supplementary file 1 [file Table_1.docx]

**PUBMED research strategy**

| Search number | Query | Search Details | Results |
| --- | --- | --- | --- |
| # 1 | "Stroke"[Mesh] | "Stroke"[MeSH Terms] | 181,230 |
| #2 | (((((((stroke[Title/Abstract]) OR (brain infarct[Title/Abstract])) OR (cerebral hemorrhage[Title/Abstract])) OR (cerebral ischemia[Title/Abstract])) OR (Cerebrovascular Accident[Title/Abstract])) OR (brain hemorrhage[Title/Abstract])) OR (Acute Ischemic Stroke[Title/Abstract])) OR (Ischemic Stroke[Title/Abstract]) | "Stroke"[Title/Abstract] OR "brain infarct"[Title/Abstract] OR "cerebral hemorrhage"[Title/Abstract] OR "cerebral ischemia"[Title/Abstract] OR "cerebrovascular accident"[Title/Abstract] OR "brain hemorrhage"[Title/Abstract] OR "acute ischemic stroke"[Title/Abstract] OR "ischemic stroke"[Title/Abstract] | 358,390 |
| #3 | "Cognitive Dysfunction"[Mesh] | "Cognitive Dysfunction"[MeSH Terms] | 40,467 |
| #4 | ((Cognitive Dysfunction[Title/Abstract]) OR (cognitive decline[Title/Abstract])) OR (cognition disorder[Title/Abstract]) | "cognitive dysfunction"[Title/Abstract] OR "cognitive decline"[Title/Abstract] OR "cognition disorder"[Title/Abstract] | 55,331 |
| #5 | #1 OR # 2 | "Stroke"[MeSH Terms] OR "Stroke"[Title/Abstract] OR "brain infarct"[Title/Abstract] OR "cerebral hemorrhage"[Title/Abstract] OR "cerebral ischemia"[Title/Abstract] OR "cerebrovascular accident"[Title/Abstract] OR "brain hemorrhage"[Title/Abstract] OR "acute ischemic stroke"[Title/Abstract] OR "ischemic stroke"[Title/Abstract] | 395,252 |
| #6 | #3 OR # 4 | "Cognitive Dysfunction"[MeSH Terms] OR "Cognitive Dysfunction"[Title/Abstract] OR "cognitive decline"[Title/Abstract] OR "cognition disorder"[Title/Abstract] | 82,197 |
| #7 | (((((prediction model[Title/Abstract]) OR (prediction tool[Title/Abstract])) OR (prognostic model[Title/Abstract])) OR (risk stratification model[Title/Abstract])) OR (model[Title/Abstract])) OR (risk assessment[Title/Abstract]) | "prediction model"[Title/Abstract] OR "prediction tool"[Title/Abstract] OR "prognostic model"[Title/Abstract] OR "risk stratification model"[Title/Abstract] OR "model"[Title/Abstract] OR "risk assessment"[Title/Abstract] | 2,961,225 |
| #8 | #5 AND # 6 AND #7 |  | 683 |
